# 7-[(3-Chloro-6-methyl-6,11-dihydro­dibenzo[*c*,*f*][1,2]thia­zepin-11-yl)amino]­hepta­noic acid *S*,*S*-dioxide hydro­chloride

**DOI:** 10.1107/S1600536812042432

**Published:** 2012-10-13

**Authors:** Anatoly Mishnev, Alvis Zvirgzdins, Andris Actins, Mara Delina

**Affiliations:** a Latvian Institute of Organic Synthesis, Aizkraukles Street 21, Riga, LV-1006, Latvia; b University of Latvia, Department of Chemistry, Kr. Valdemara Street 48, Riga, LV-1013, Latvia

## Abstract

In the title compound, C_21_H_26_ClN_2_O_4_S^.^Cl, also known as tianeptine hydro­chloride, the seven-membered ring adopts a boat conformation. The dihedral angle between the mean planes of the benzene rings is 44.44 (7)°. There is an intra­molecular hydrogen bond formed *via* S= O⋯H—N. In the crystal, mol­ecules are connected *via* pairs of N—H.·O, N—H⋯Cl and O—H⋯Cl hydrogen bonds, forming inversion dimers, which are consolidated by C—H⋯O inter­actions. The dimers are linked by C—H⋯O and C—H⋯Cl inter­actions, forming a two-dimensional network lying parallel to (011).

## Related literature

For general information about tianeptine and its preparation, see: Guzman *et al.* (2010 ▶). For related structures, see: Orola *et al.* (2012 ▶).
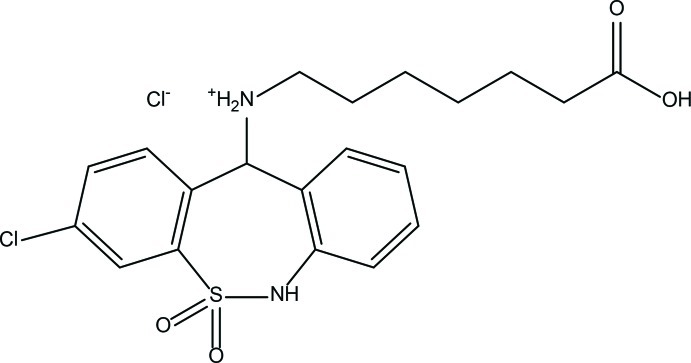


## Experimental

### 

#### Crystal data

C_21_H_26_ClN_2_O_4_S^+^·Cl^−^
*M* *_r_* = 473.40
Triclinic, 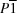

*a* = 9.5439 (2) Å
*b* = 10.0910 (2) Å
*c* = 13.1802 (3) Å
α = 104.000 (1)°
β = 101.538 (1)°
γ = 105.018 (1)°
*V* = 1139.04 (4) Å^3^
*Z* = 2
Mo *K*α radiation
μ = 0.41 mm^−1^
*T* = 190 K
0.24 × 0.20 × 0.14 mm

#### Data collection

Nonius KappaCCD diffractometer
7552 measured reflections
4985 independent reflections
4015 reflections with *I* > 2σ(*I*)
*R*_int_ = 0.021

#### Refinement

*R*[*F* ^2^ > 2σ(*F* ^2^)] = 0.040
*wR*(*F* ^2^) = 0.100
*S* = 1.03
4985 reflections
273 parameters
H-atom parameters constrained
Δρ_max_ = 0.76 e Å^−3^
Δρ_min_ = −0.34 e Å^−3^

### 

Data collection: *COLLECT* (Hooft, 1998 ▶); cell refinement: *HKL* *DENZO* (Otwinowski & Minor, 1997 ▶); data reduction: *SCALEPACK* (Otwinowski & Minor, 1997 ▶); program(s) used to solve structure: *SHELXS97* (Sheldrick, 2008 ▶); program(s) used to refine structure: *SHELXL97* (Sheldrick, 2008 ▶); molecular graphics: *Mercury* (Macrae *et al.*, 2008 ▶); software used to prepare material for publication: *SHELXL97* and *publCIF* (Westrip, 2010 ▶).

## Supplementary Material

Crystal structure: contains datablock(s) global. DOI: 10.1107/S1600536812042432/pv2586sup1.cif

Additional supplementary materials:  crystallographic information; 3D view; checkCIF report

## Figures and Tables

**Table 1:** Hydrogen-bond geometry (Å, °)

*D*—H⋯*A*	*D*—H	H⋯*A*	*D*⋯*A*	*D*—H⋯*A*
N2—H3*A*⋯Cl2^i^	0.90	2.31	3.154 (2)	157
N2—H3*B*⋯O3^ii^	0.90	2.32	2.821 (2)	115
C16—H11*B*⋯O3^ii^	0.97	2.56	3.201 (2)	124
O4—H6⋯Cl2^iii^	0.82	2.22	3.043 (2)	176
C4—H3⋯Cl2^iv^	0.93	2.82	3.651 (2)	150
C18—H6*A*⋯O4^v^	0.97	2.56	3.467 (2)	157
C7—H7⋯Cl2	0.98	2.59	3.534 (2)	162
N2—H3*B*⋯O2	0.90	2.02	2.802 (2)	144

Symmetry codes: (i) 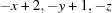; (ii) 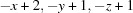; (iii) 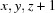; (iv) 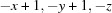; (v) 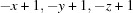.

**Fig. 1.**
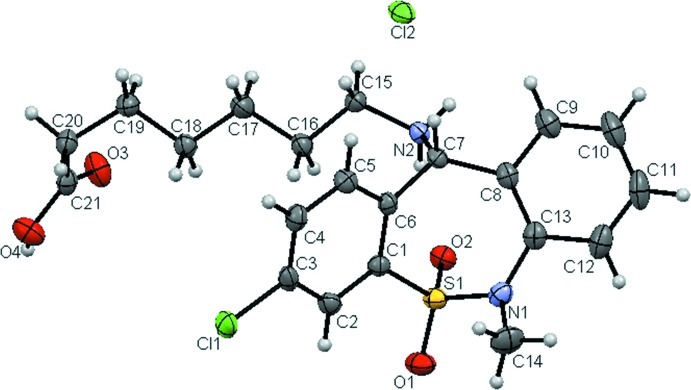
The molecular structure of the title compound showing 50% probability ellipsoids and hydrogen atoms are shown as small spheres of arbitrary radii.

**Fig. 2.**
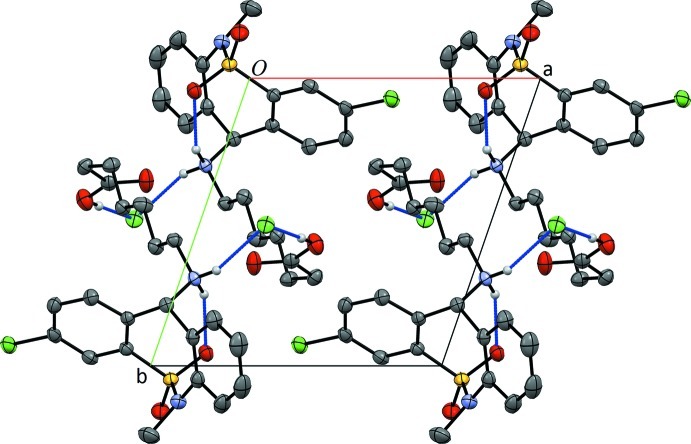
Packing diagram of the title compound viewed along the c axis. Blue lines indicate hydrogen bonds.

## supplementary crystallographic information

### Comment

Tianeptine salts are of wide interest since they are crystalline. Although the synthesis of the title compound, tianeptine hydrochloride, has been described (Guzman *et al.*, 2010) we describe in this article an improved method of its synthesis and its crystal structure.

In the title compound (Fig. 1), the seven-membered ring adopts a boat conformation with the values of torsion angles: C1—S1—N1—C13 = -78.4 (2) and C1—C6—C7—C8 = -56.3 (3)°. The dihedral angle between the mean planes of the two benzene rings (C1–C6 and C8–C13) is 44.44 (7)°. There is an intramolecular hydrogen bond in the title molecule which is formed *via* S1═O2···H3B—N2 that stabilizes the molecular structure. In the crystal, the molecules are connected *via* hydrogen bonds between carboxyl and amine groups and chloride anion, O4—H6···Cl2, N2—H3A···Cl2 and N2—H3B···O3. The crystal structure is further consolidated by intermolecular interactions, C18—H6A···O4, C4—H3···Cl2, C16—H11B···O3 and C7—H7···Cl2 (Table 1 and Fig. 2). The supramolecular structure of tianeptine hydrochloride consists of parallel oriented tricyclic fragment and parallel oriented carbon atom chains (heptanoic acid). Carbon atom chains are linked with hydrogen bonds *via* chloride anion, amine and carboxylgroup. The torsion angle C8—C7—N2—C15 is -168.4 (2)° so that the carbon atom chain C15—C20 is almost parallel to the benzene ring C8—C13.

The crystal structures of tianeptine polymorphs have been reported recently (Orola *et al.*, 2012). The title structure is more similar with polymorph A structure in which tianeptine molecules are linked *via* hydrogen bonds between amine and carboxyl groups. The tianeptine molecules in the structure of tianeptine polymorph B are in a zwiterrion form.

### Experimental

Tianeptine sodium salt (0.5 g;1.09 mmol) was dissolved in 20 ml deionized water in a Erlenmeyer flask and added ~3 mmol of hydrochloric acid. Mixture were stirred for 6 h. After 6 h suspension was filtered and washed with cold water. The product was dried and recrystallized from water by slow evaporation at room temperature.

### Refinement

All hydrogen atoms were positioned geometrically with C—H distances ranging from 0.93 to 0.97 Å and refined as riding on their parent atoms with *U*_iso_(H) = 1.5 *U*_eq_(C) for methyl groups and *U*_iso_(H) = 1.2 *U*_eq_(C) for others.

### Crystal data

**Table:**

C_21_H_26_ClN_2_O_4_S^+^·Cl^−^	*Z* = 2
*M**_r_* = 473.40	*F*(000) = 496
Triclinic, *P*1	*D*_x_ = 1.380 Mg m^−^^3^
Hall symbol: -P 1	Mo *K*α radiation, λ = 0.71073 Å
*a* = 9.5439 (2) Å	Cell parameters from 6759 reflections
*b* = 10.0910 (2) Å	θ = 1.0–27.1°
*c* = 13.1802 (3) Å	µ = 0.41 mm^−^^1^
α = 104.4000 (12)°	*T* = 190 K
β = 101.538 (1)°	Plate, colourless
γ = 105.0180 (11)°	0.24 × 0.20 × 0.14 mm
*V* = 1139.04 (4) Å^3^	

### Data collection

**Table:**

Nonius KappaCCD diffractometer	4015 reflections with *I* > 2σ(*I*)
Radiation source: fine-focus sealed tube	*R*_int_ = 0.021
Graphite monochromator	θ_max_ = 27.1°, θ_min_ = 3.1°
CCD scans	*h* = −11→12
7552 measured reflections	*k* = −12→12
4985 independent reflections	*l* = −16→16

### Refinement

**Table:**

Refinement on *F*^2^	Primary atom site location: structure-invariant direct methods
Least-squares matrix: full	Secondary atom site location: difference Fourier map
*R*[*F*^2^ > 2σ(*F*^2^)] = 0.040	Hydrogen site location: inferred from neighbouring sites
*wR*(*F*^2^) = 0.100	H-atom parameters constrained
*S* = 1.03	*w* = 1/[σ^2^(*F*_o_^2^) + (0.0363*P*)^2^ + 0.641*P*] where *P* = (*F*_o_^2^ + 2*F*_c_^2^)/3
4985 reflections	(Δ/σ)_max_ = 0.018
273 parameters	Δρ_max_ = 0.76 e Å^−^^3^
0 restraints	Δρ_min_ = −0.34 e Å^−^^3^

### Special details

**Table:**

Geometry. All e.s.d.'s (except the e.s.d. in the dihedral angle between two l.s. planes) are estimated using the full covariance matrix. The cell e.s.d.'s are taken into account individually in the estimation of e.s.d.'s in distances, angles and torsion angles; correlations between e.s.d.'s in cell parameters are only used when they are defined by crystal symmetry. An approximate (isotropic) treatment of cell e.s.d.'s is used for estimating e.s.d.'s involving l.s. planes.
Refinement. Refinement of *F*^2^ against ALL reflections. The weighted *R*-factor *wR* and goodness of fit *S* are based on *F*^2^, conventional *R*-factors *R* are based on *F*, with *F* set to zero for negative *F*^2^. The threshold expression of *F*^2^ > σ(*F*^2^) is used only for calculating *R*-factors(gt) *etc*. and is not relevant to the choice of reflections for refinement. *R*-factors based on *F*^2^ are statistically about twice as large as those based on *F*, and *R*- factors based on ALL data will be even larger.

### Fractional atomic coordinates and isotropic or equivalent isotropic displacement parameters (Å^2^)

**Table:**

	*x*	*y*	*z*	*U*_iso_*/*U*_eq_	
Cl1	0.48761 (6)	0.92769 (6)	0.32682 (5)	0.03721 (15)	
S1	1.08561 (5)	1.04778 (5)	0.36222 (4)	0.02568 (12)	
Cl2	0.77093 (5)	0.48993 (5)	−0.09234 (4)	0.03147 (13)	
O2	1.17517 (14)	0.95570 (14)	0.37600 (11)	0.0274 (3)	
N2	1.04759 (17)	0.69630 (16)	0.20172 (13)	0.0218 (3)	
H3A	1.1139	0.6682	0.1689	0.026*	
H3B	1.0994	0.7516	0.2708	0.026*	
O4	0.57739 (17)	0.42297 (18)	0.67606 (12)	0.0375 (4)	
H6	0.6331	0.4443	0.7378	0.056*	
O1	1.08744 (17)	1.15746 (16)	0.45495 (12)	0.0371 (4)	
O3	0.76590 (16)	0.35639 (18)	0.62834 (12)	0.0382 (4)	
N1	1.13800 (18)	1.12491 (17)	0.27471 (14)	0.0281 (4)	
C5	0.7141 (2)	0.7622 (2)	0.13441 (16)	0.0264 (4)	
H2	0.6903	0.6926	0.0667	0.032*	
C4	0.5978 (2)	0.7900 (2)	0.17507 (17)	0.0288 (4)	
H3	0.4974	0.7398	0.1352	0.035*	
C6	0.8655 (2)	0.83537 (19)	0.19193 (15)	0.0212 (4)	
C2	0.7818 (2)	0.9710 (2)	0.33545 (16)	0.0265 (4)	
H5	0.8047	1.0416	0.4025	0.032*	
C18	0.7174 (2)	0.4501 (2)	0.40383 (16)	0.0266 (4)	
H6A	0.6521	0.5092	0.4011	0.032*	
H6B	0.7988	0.4970	0.4713	0.032*	
C7	0.9826 (2)	0.7887 (2)	0.14329 (15)	0.0216 (4)	
H7	0.9253	0.7226	0.0698	0.026*	
C3	0.6328 (2)	0.8933 (2)	0.27553 (17)	0.0272 (4)	
C21	0.6408 (2)	0.3647 (2)	0.60345 (16)	0.0261 (4)	
C20	0.5396 (2)	0.3075 (2)	0.48869 (16)	0.0282 (4)	
H10A	0.4775	0.3685	0.4796	0.034*	
H10B	0.4726	0.2109	0.4761	0.034*	
C16	0.8769 (2)	0.5855 (2)	0.30509 (16)	0.0268 (4)	
H11A	0.8158	0.6486	0.3035	0.032*	
H11B	0.9622	0.6312	0.3702	0.032*	
C1	0.8963 (2)	0.9404 (2)	0.29264 (15)	0.0228 (4)	
C15	0.9334 (2)	0.5636 (2)	0.20475 (16)	0.0249 (4)	
H13A	0.9785	0.4873	0.2007	0.030*	
H13B	0.8475	0.5310	0.1405	0.030*	
C13	1.1778 (2)	1.0475 (2)	0.18368 (16)	0.0273 (4)	
C19	0.6274 (2)	0.3021 (2)	0.40408 (16)	0.0291 (4)	
H15A	0.6963	0.2486	0.4177	0.035*	
H15B	0.5570	0.2497	0.3322	0.035*	
C8	1.1112 (2)	0.8998 (2)	0.12618 (15)	0.0244 (4)	
C17	0.7835 (3)	0.4409 (2)	0.30818 (18)	0.0338 (5)	
H17A	0.7014	0.3949	0.2411	0.041*	
H17B	0.8465	0.3796	0.3104	0.041*	
C9	1.1641 (2)	0.8421 (3)	0.03912 (17)	0.0338 (5)	
H18	1.1217	0.7438	0.0004	0.041*	
C14	1.0767 (3)	1.2418 (2)	0.2609 (2)	0.0444 (6)	
H19A	1.1297	1.2932	0.2203	0.067*	
H19B	1.0895	1.3074	0.3313	0.067*	
H19C	0.9710	1.2007	0.2221	0.067*	
C12	1.2910 (2)	1.1316 (3)	0.1514 (2)	0.0381 (5)	
H20	1.3341	1.2301	0.1891	0.046*	
C10	1.2774 (3)	0.9263 (3)	0.0089 (2)	0.0437 (6)	
H21	1.3110	0.8851	−0.0489	0.052*	
C11	1.3399 (3)	1.0725 (3)	0.0656 (2)	0.0437 (6)	
H22	1.4151	1.1306	0.0455	0.052*	

### Atomic displacement parameters (Å^2^)

**Table:**

	*U*^11^	*U*^22^	*U*^33^	*U*^12^	*U*^13^	*U*^23^
Cl1	0.0319 (3)	0.0313 (3)	0.0597 (4)	0.0146 (2)	0.0278 (3)	0.0173 (3)
S1	0.0248 (2)	0.0244 (2)	0.0245 (2)	0.00797 (19)	0.00501 (19)	0.0035 (2)
Cl2	0.0313 (3)	0.0342 (3)	0.0234 (2)	0.0100 (2)	0.0077 (2)	0.0005 (2)
O2	0.0264 (7)	0.0312 (7)	0.0254 (7)	0.0130 (6)	0.0048 (6)	0.0086 (6)
N2	0.0221 (8)	0.0241 (8)	0.0221 (8)	0.0099 (6)	0.0091 (6)	0.0075 (7)
O4	0.0396 (9)	0.0494 (9)	0.0282 (8)	0.0243 (8)	0.0087 (7)	0.0107 (7)
O1	0.0359 (8)	0.0334 (8)	0.0324 (8)	0.0106 (7)	0.0060 (6)	−0.0025 (7)
O3	0.0293 (8)	0.0611 (10)	0.0343 (8)	0.0210 (7)	0.0112 (6)	0.0242 (8)
N1	0.0265 (8)	0.0238 (8)	0.0337 (9)	0.0080 (7)	0.0070 (7)	0.0102 (7)
C5	0.0272 (10)	0.0288 (10)	0.0235 (10)	0.0096 (8)	0.0062 (8)	0.0091 (8)
C4	0.0223 (10)	0.0304 (11)	0.0353 (11)	0.0078 (8)	0.0088 (8)	0.0132 (9)
C6	0.0222 (9)	0.0227 (9)	0.0225 (9)	0.0088 (7)	0.0083 (7)	0.0108 (8)
C2	0.0321 (10)	0.0236 (10)	0.0290 (10)	0.0129 (8)	0.0132 (8)	0.0096 (8)
C18	0.0287 (10)	0.0273 (10)	0.0288 (10)	0.0114 (8)	0.0123 (8)	0.0119 (9)
C7	0.0234 (9)	0.0242 (9)	0.0180 (9)	0.0085 (7)	0.0065 (7)	0.0068 (8)
C3	0.0277 (10)	0.0262 (10)	0.0398 (11)	0.0140 (8)	0.0192 (9)	0.0179 (9)
C21	0.0273 (10)	0.0261 (10)	0.0300 (10)	0.0086 (8)	0.0106 (8)	0.0160 (9)
C20	0.0254 (10)	0.0293 (10)	0.0291 (10)	0.0047 (8)	0.0082 (8)	0.0119 (9)
C16	0.0299 (10)	0.0284 (10)	0.0264 (10)	0.0109 (8)	0.0135 (8)	0.0103 (9)
C1	0.0230 (9)	0.0219 (9)	0.0249 (9)	0.0086 (7)	0.0072 (7)	0.0086 (8)
C15	0.0274 (10)	0.0221 (9)	0.0271 (10)	0.0081 (8)	0.0112 (8)	0.0083 (8)
C13	0.0233 (10)	0.0323 (11)	0.0311 (10)	0.0111 (8)	0.0076 (8)	0.0163 (9)
C19	0.0324 (11)	0.0262 (10)	0.0272 (10)	0.0064 (8)	0.0107 (8)	0.0074 (9)
C8	0.0227 (9)	0.0314 (10)	0.0235 (9)	0.0114 (8)	0.0072 (8)	0.0133 (8)
C17	0.0452 (12)	0.0280 (11)	0.0340 (11)	0.0132 (9)	0.0209 (10)	0.0105 (9)
C9	0.0341 (11)	0.0434 (12)	0.0309 (11)	0.0161 (10)	0.0141 (9)	0.0162 (10)
C14	0.0553 (15)	0.0302 (12)	0.0531 (15)	0.0198 (11)	0.0141 (12)	0.0171 (11)
C12	0.0286 (11)	0.0400 (13)	0.0507 (14)	0.0077 (9)	0.0107 (10)	0.0270 (12)
C10	0.0401 (13)	0.0665 (17)	0.0386 (13)	0.0212 (12)	0.0233 (11)	0.0272 (13)
C11	0.0323 (12)	0.0622 (17)	0.0528 (15)	0.0146 (11)	0.0216 (11)	0.0391 (14)

### Geometric parameters (Å, º)

**Table:**

Cl1—C3	1.7332 (19)	C7—H7	0.9800
S1—O1	1.4240 (15)	C21—C20	1.499 (3)
S1—O2	1.4354 (14)	C20—C19	1.522 (3)
S1—N1	1.6315 (17)	C20—H10A	0.9700
S1—C1	1.7629 (19)	C20—H10B	0.9700
N2—C15	1.507 (2)	C16—C15	1.513 (3)
N2—C7	1.521 (2)	C16—C17	1.515 (3)
N2—H3A	0.9000	C16—H11A	0.9700
N2—H3B	0.9000	C16—H11B	0.9700
O4—C21	1.328 (2)	C15—H13A	0.9700
O4—H6	0.8200	C15—H13B	0.9700
O3—C21	1.204 (2)	C13—C8	1.397 (3)
N1—C13	1.436 (3)	C13—C12	1.400 (3)
N1—C14	1.480 (3)	C19—H15A	0.9700
C5—C4	1.387 (3)	C19—H15B	0.9700
C5—C6	1.392 (3)	C8—C9	1.401 (3)
C5—H2	0.9300	C17—H17A	0.9700
C4—C3	1.381 (3)	C17—H17B	0.9700
C4—H3	0.9300	C9—C10	1.384 (3)
C6—C1	1.398 (3)	C9—H18	0.9300
C6—C7	1.513 (2)	C14—H19A	0.9600
C2—C3	1.388 (3)	C14—H19B	0.9600
C2—C1	1.393 (3)	C14—H19C	0.9600
C2—H5	0.9300	C12—C11	1.369 (3)
C18—C17	1.512 (3)	C12—H20	0.9300
C18—C19	1.518 (3)	C10—C11	1.381 (4)
C18—H6A	0.9700	C10—H21	0.9300
C18—H6B	0.9700	C11—H22	0.9300
C7—C8	1.529 (2)		
			
O1—S1—O2	119.59 (9)	C15—C16—C17	109.79 (16)
O1—S1—N1	108.15 (9)	C15—C16—H11A	109.7
O2—S1—N1	106.88 (8)	C17—C16—H11A	109.7
O1—S1—C1	108.63 (9)	C15—C16—H11B	109.7
O2—S1—C1	109.38 (8)	C17—C16—H11B	109.7
N1—S1—C1	102.91 (8)	H11A—C16—H11B	108.2
C15—N2—C7	115.42 (14)	C2—C1—C6	122.19 (17)
C15—N2—H3A	108.4	C2—C1—S1	118.86 (15)
C7—N2—H3A	108.4	C6—C1—S1	118.74 (14)
C15—N2—H3B	108.4	N2—C15—C16	114.61 (16)
C7—N2—H3B	108.4	N2—C15—H13A	108.6
H3A—N2—H3B	107.5	C16—C15—H13A	108.6
C21—O4—H6	109.5	N2—C15—H13B	108.6
C13—N1—C14	117.16 (17)	C16—C15—H13B	108.6
C13—N1—S1	120.96 (13)	H13A—C15—H13B	107.6
C14—N1—S1	115.98 (15)	C8—C13—C12	119.4 (2)
C4—C5—C6	121.92 (19)	C8—C13—N1	125.42 (17)
C4—C5—H2	119.0	C12—C13—N1	115.16 (19)
C6—C5—H2	119.0	C18—C19—C20	113.90 (17)
C3—C4—C5	119.18 (18)	C18—C19—H15A	108.8
C3—C4—H3	120.4	C20—C19—H15A	108.8
C5—C4—H3	120.4	C18—C19—H15B	108.8
C5—C6—C1	117.17 (17)	C20—C19—H15B	108.8
C5—C6—C7	117.27 (17)	H15A—C19—H15B	107.7
C1—C6—C7	125.46 (16)	C13—C8—C9	117.75 (18)
C3—C2—C1	118.31 (18)	C13—C8—C7	128.70 (17)
C3—C2—H5	120.8	C9—C8—C7	113.54 (18)
C1—C2—H5	120.8	C18—C17—C16	114.47 (17)
C17—C18—C19	112.27 (17)	C18—C17—H17A	108.6
C17—C18—H6A	109.2	C16—C17—H17A	108.6
C19—C18—H6A	109.2	C18—C17—H17B	108.6
C17—C18—H6B	109.2	C16—C17—H17B	108.6
C19—C18—H6B	109.2	H17A—C17—H17B	107.6
H6A—C18—H6B	107.9	C10—C9—C8	122.2 (2)
C6—C7—N2	111.23 (14)	C10—C9—H18	118.9
C6—C7—C8	120.45 (16)	C8—C9—H18	118.9
N2—C7—C8	109.32 (14)	N1—C14—H19A	109.5
C6—C7—H7	104.8	N1—C14—H19B	109.5
N2—C7—H7	104.8	H19A—C14—H19B	109.5
C8—C7—H7	104.8	N1—C14—H19C	109.5
C4—C3—C2	121.21 (18)	H19A—C14—H19C	109.5
C4—C3—Cl1	119.27 (15)	H19B—C14—H19C	109.5
C2—C3—Cl1	119.51 (16)	C11—C12—C13	121.6 (2)
O3—C21—O4	122.96 (19)	C11—C12—H20	119.2
O3—C21—C20	123.80 (19)	C13—C12—H20	119.2
O4—C21—C20	113.22 (17)	C11—C10—C9	119.1 (2)
C21—C20—C19	112.61 (16)	C11—C10—H21	120.4
C21—C20—H10A	109.1	C9—C10—H21	120.4
C19—C20—H10A	109.1	C12—C11—C10	119.9 (2)
C21—C20—H10B	109.1	C12—C11—H22	120.0
C19—C20—H10B	109.1	C10—C11—H22	120.0
H10A—C20—H10B	107.8		
			
O1—S1—N1—C13	166.82 (14)	N1—S1—C1—C2	−116.33 (15)
O2—S1—N1—C13	36.83 (17)	O1—S1—C1—C6	173.00 (14)
C1—S1—N1—C13	−78.34 (16)	O2—S1—C1—C6	−54.86 (16)
O1—S1—N1—C14	−41.01 (17)	N1—S1—C1—C6	58.50 (16)
O2—S1—N1—C14	−170.99 (15)	C7—N2—C15—C16	−92.52 (19)
C1—S1—N1—C14	73.83 (17)	C17—C16—C15—N2	−170.85 (16)
C6—C5—C4—C3	0.0 (3)	C14—N1—C13—C8	−117.4 (2)
C4—C5—C6—C1	−0.8 (3)	S1—N1—C13—C8	34.5 (3)
C4—C5—C6—C7	175.82 (17)	C14—N1—C13—C12	61.3 (2)
C5—C6—C7—N2	−102.80 (18)	S1—N1—C13—C12	−146.80 (15)
C1—C6—C7—N2	73.5 (2)	C17—C18—C19—C20	−171.27 (17)
C5—C6—C7—C8	127.40 (18)	C21—C20—C19—C18	−68.0 (2)
C1—C6—C7—C8	−56.3 (2)	C12—C13—C8—C9	1.3 (3)
C15—N2—C7—C6	56.1 (2)	N1—C13—C8—C9	179.94 (17)
C15—N2—C7—C8	−168.43 (15)	C12—C13—C8—C7	−177.35 (18)
C5—C4—C3—C2	1.0 (3)	N1—C13—C8—C7	1.3 (3)
C5—C4—C3—Cl1	−179.69 (14)	C6—C7—C8—C13	27.4 (3)
C1—C2—C3—C4	−1.2 (3)	N2—C7—C8—C13	−103.2 (2)
C1—C2—C3—Cl1	179.56 (13)	C6—C7—C8—C9	−151.28 (17)
O3—C21—C20—C19	−26.4 (3)	N2—C7—C8—C9	78.08 (19)
O4—C21—C20—C19	155.14 (17)	C19—C18—C17—C16	−178.95 (17)
C3—C2—C1—C6	0.3 (3)	C15—C16—C17—C18	−178.47 (17)
C3—C2—C1—S1	174.95 (13)	C13—C8—C9—C10	−0.6 (3)
C5—C6—C1—C2	0.7 (3)	C7—C8—C9—C10	178.25 (19)
C7—C6—C1—C2	−175.65 (17)	C8—C13—C12—C11	−0.9 (3)
C5—C6—C1—S1	−174.00 (13)	N1—C13—C12—C11	−179.73 (19)
C7—C6—C1—S1	9.7 (2)	C8—C9—C10—C11	−0.5 (3)
O1—S1—C1—C2	−1.84 (17)	C13—C12—C11—C10	−0.2 (3)
O2—S1—C1—C2	130.31 (15)	C9—C10—C11—C12	0.9 (3)

### Hydrogen-bond geometry (Å, º)

**Table:**

*D*—H···*A*	*D*—H	H···*A*	*D*···*A*	*D*—H···*A*
N2—H3*A*···Cl2^i^	0.90	2.31	3.154 (2)	157
N2—H3*B*···O3^ii^	0.90	2.32	2.821 (2)	115
C16—H11*B*···O3^ii^	0.97	2.56	3.201 (2)	124
O4—H6···Cl2^iii^	0.82	2.22	3.043 (2)	176
C4—H3···Cl2^iv^	0.93	2.82	3.651 (2)	150
C18—H6*A*···O4^v^	0.97	2.56	3.467 (2)	157
C7—H7···Cl2	0.98	2.59	3.534 (2)	162
N2—H3*B*···S1	0.90	2.98	3.533 (2)	121
N2—H3*B*···O2	0.90	2.02	2.802 (2)	144
C2—H5···O1	0.93	2.52	2.894 (2)	104
C14—H19*B*···O1	0.96	2.48	2.881 (3)	105

Symmetry codes: (i) −*x*+2, −*y*+1, −*z*; (ii) −*x*+2, −*y*+1, −*z*+1; (iii) *x*, *y*, *z*+1; (iv) −*x*+1, −*y*+1, −*z*; (v) −*x*+1, −*y*+1, −*z*+1.
